# Identification of a Differential Microbiological Signature of Extended‐Spectrum ß‐Lactamases Producing *Escherichia* Carriers in Spanish Community Children

**DOI:** 10.1002/mbo3.70077

**Published:** 2025-10-29

**Authors:** Martina Cardinali‐Benigni, Olfat Khannous‐Lleiffe, Ester Saus, David Carmena, Michael J. McConnell, Toni Gabaldón, Mireia López‐Siles

**Affiliations:** ^1^ Institute for Research in Biomedicine (IRB Barcelona) The Barcelona Institute of Science and Technology Barcelona Spain; ^2^ Barcelona Supercomputing Centre (BSC‐CNS) Institute for Research in Biomedicine (IRB) Barcelona Spain; ^3^ Centre for Biomedical Reseach Network (CIBER) in Infectious Diseases Health Institute Carlos III (ISCIII) Madrid Spain; ^4^ Universitat de Barcelona Barcelona Spain; ^5^ Parasitology Reference and Research Laboratory, National Centre for Microbiology Health Institute Carlos III (ISCIII) Majadahonda Spain; ^6^ Department of Biological Sciences University of Notre Dame Notre Dame IN USA; ^7^ ICREA Barcelona Spain; ^8^ Microbiology of Intestinal Diseases, Biology Department Universitat de Girona Girona Spain

**Keywords:** children, *Escherichia*, extended‐spectrum ß‐lactamase, microbiota, multidrug resistance

## Abstract

Gut colonization by Extended‐spectrum ß‐lactamase‐producing Enterobacteriaceae (ESBL‐E), is increasing among nonhospitalized children, but the impact of ESBL‐E carriage on the intestinal microbiota remains unknown. In this study, we compared the fecal bacterial composition in 24 children (3–11 years old) that were carriers of *Escherichia* producing ESBL (ESBL‐Es) to that of an age‐ and gender‐matched group of non‐carriers using 16S rRNA gene amplicon sequencing. Alpha diversity of the community was similar in both groups. No significant differences in relative abundance at the phylum, class, and order levels were found. Significant differences at the genus and species level were identified. The genus *Blautia* and the species *Blautia obeum* were more abundant in non‐carriers, whereas the genus *Coprococcus* and *Coprococcus eutactus* species were more abundant in carriers. Generalized linear models, confirmed these differences and identified additional ones at the family (Tannerellaceae is more abundant in carries), genus (*Parabacteroides* is more abundant in carriers), and species (*Bifidobacterium longum* and *Ruminococcus gauvreauii* are more abundant in non‐carriers, and *Parabacteroides distasonis* in carriers) levels. However, statistical significance was lost in all cases after applying multiple testing correction. Altogether, this study uncovers a differential microbiological signature between pediatric ESBL‐Es carriers and non‐carriers, despite an overall similar diversity and composition of their gut microbiota. Further studies are required to explore if these differences predispose to, or are a consequence of ESBL‐Es carriage, and if modulation of the gut microbiota community may help to prevent or eliminate ESBL‐Es colonization.

## Graphical abstract

This study provides evidence that colonization by Extended spectrum β‐lactamase‐*Escherichia coli* in children does not have major impact in gut bacterial community diversity although differences in composition at genus and species level exist.

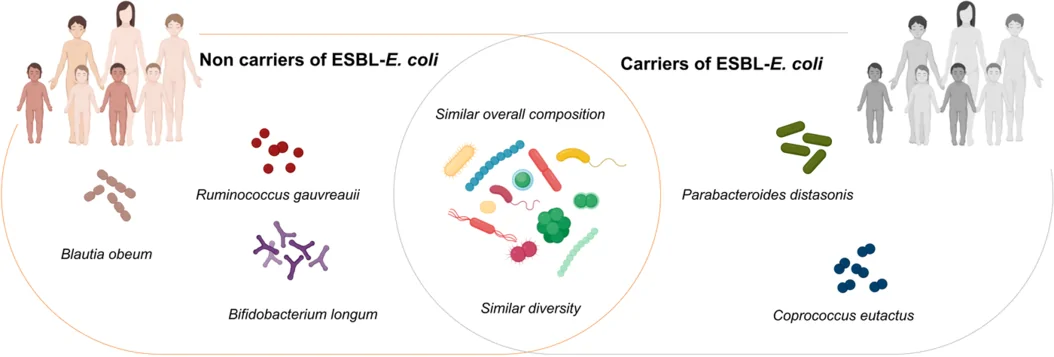

## 1 Background

Antibiotic resistance increases patients' recovery time, mortality, and negatively impacts healthcare costs (Pitout and Laupland 2008). Extended Spectrum ß‐lactamases (ESBL) producing Enterobacteriaceae (ESBL‐E), mainly involving *Escherichia* and *Klebsiella* genus, have been identified by the World Health Organization (WHO) among priority pathogens for which new antimicrobials are urgently needed (Tacconelli et al. 2018). Firstly, because the number of effective antimicrobials available for treating these infections is dramatically reduced. Particularly, microorganisms producing ESBL are resistant to ß‐lactams, including cephalosporins, and concomitant carriage of other resistance genes has been reported (Pitout and Laupland 2008; Coque et al. 2008; Guimarães et al. 2009; Rodríguez‐Revuelta et al. 2018). Secondly, over the past two decades, ESBL‐E carriage has considerably increased both in clinical and community settings, including pediatric populations (Bezabih et al. 2022; Lukac et al. 2015).

According to the Antibiotic Resistance Threats Report published by the Centers for Disease Control and Prevention in 2019 almost half of ESBL‐E infections were community‐associated (CDC 2019). ESBL‐E isolated from infections and the fecal ESBL‐E colonization rate in the community varies among countries (Coque et al. 2008; Woerther et al. 2013). Specifically, in cohorts of Spanish children, colonization rates range between 2.6%–24% (Guimarães et al. 2009; Rodríguez‐Revuelta et al. 2018; Bezabih et al. 2022; López‐Siles et al. 2023). Thus, in certain populations it may be greater than the average rate of ESBL‐E carriers among healthy children in Europe, which has been reported as less than 5% (Lukac et al. 2015; CDC 2019; Woerther et al. 2013).

The gut microbiota of healthy individuals represents a reservoir for ESBL‐E (Woerther et al. 2013), and plays a role in transmission and spread (Fernández‐Reyes et al. 2014). Another concern is that when present in the gut, these bacteria might cause infections or transfer their resistance genes to other bacteria in the gut. Intestinal microbiota may prevent pathogens colonization by releasing inhibitory substances, competing for nutrients or changing the gut environment (Ducarmon et al. 2019; Kim et al. 2017). For example, anaerobic microbiota in the colon can inhibit colonization by vancomycin resistant enterococci (Pultz et al. 2005).

There is a growing interest in deciphering if the risk of colonization by resistant bacteria is dependent on our intestinal microbiota and what mechanisms could be involved. Specifically, four studies have evaluated the impact of ESBL‐E colonization on the gut microbiota. A study in healthy Amerindian adults from the French Guiana depicted bacterial genera (*Desulfovibrio*, *Oscillospira*, *Parabacteroides*, and *Coprococcus*) significantly more abundant in the active microbiota of non‐carriers compared to ESBL‐E carriers (Gosalbes et al. 2016). In a Thai cohort, *Bacteroides uniformis* was significantly less abundant in ESBL‐E carriers than in non‐carriers (Piewngam et al. 2019). A study enrolling international travelers from Hong Kong identified increased abundance of several opportunistic pathogens in ESBL‐E carriers (Peng et al. 2021). The only study in the European WHO region found no differences in bacterial diversity or composition between ESBL‐E carrier and ESBL‐E non‐carrier groups at species level (Ducarmon et al. 2022). However, to the best of our knowledge, no similar study has focused on pediatric population. Because it is well known that gut microbiota evolves throughout the human lifespan and that conditions in childhood may influence host wellbeing in adulthood, collecting information in this group of age is of utmost relevance.

The main aim of this study was to characterize the composition of the intestinal microbiota of healthy schoolchildren who were either ESBL‐*Escherichia* (ESBL‐Es) carriers or non‐carriers to investigate possible alterations associated with the presence of these multidrug‐resistant microorganisms. Microbiota and sociodemographic data were analyzed to identify risk or preventive biomarkers for colonization.

## 2 Materials and Methods

### 2.1 Study Design, Setting, and Recruitment of Volunteers

Subjects were recruited as part of a larger retrospective cross‐sectional study engaging 936 apparently healthy children (3–13 years old), attending seven public primary and secondary schools in Madrid (Reh et al. 2019). That seminal survey was originally designed to molecularly assess the asymptomatic carriage and genetic diversity of protist parasites potentially linked to gastrointestinal manifestations such as diarrhea. The associated metadata included sociodemographic (age, sex, country of birth, number of household siblings), behavioral (hygiene practices, type of drinking water, contact with pets) and clinical (history of diarrhea prior sampling) variables which could act as potential risk or protective factors for parasite infection. After the sampling campaign (April–June 2018), 887 individuals were tested for ESBL‐E intestinal carriage, as described elsewhere (López‐Siles et al. 2023). Antimicrobial susceptibility of ESBL‐E isolates to clinically relevant antibiotics was determined following EUCAST and CLSI guidelines (CLSI 2020).

Twenty‐four individuals included in the initial study (Reh et al. 2019) were identified as ESBL‐E carriers (López‐Siles et al. 2023). In all cases the ESBL‐E isolate was from the genus *Escherichia*. Specifically, 23 out of the 24 isolates were *Escherichia coli*. Characteristics of strains are shown in Supporting Table 1. Among them, six had Sulfhydryl Variable (SHV) ESBL while 18 were carrying CefoTaXime‐hydrolyzing β‐lactamase, Munich (CTX‐M) ESBL. A paired cohort of twenty‐four non‐carriers was selected from the remaining 863 children, to match in age, gender, educational center, and as close as possible to the corresponding positive subject regarding all the other sociodemographic data collected.

Sample size was considered suitable for this exploratory approach considering that previous studies aiming to establish microbiota involvement in ESBL‐E carriers (Gosalbes et al. 2016; Piewngam et al. 2019; Peng et al. 2021; Ducarmon et al. 2022) were performed with similar sample sizes.

### 2.2 Sample, Metadata Collection and DNA Extraction

One stool sample and a questionnaire per participant were submitted. Feces were collected by the parents/legal guardians of children after appropriate training. The fecal samples were refrigerated immediately after collection. Within the 24 h of defecation, the samples were taken to the Spanish National Centre for Microbiology and stored long‐term at ‒80°C without stabilizers until analysis. A standardized epidemiological questionnaire was used to collect demographic characteristics, behavioral habits, and additional information on potential risk/protective factors for protozoan enteroparasites colonization/infection. Information on the occurrence of enteric protists, as established through molecular methods, has been published elsewhere (Reh et al. 2019). In addition, consistency of feces was assessed using the Bristol stool scale (Lewis and Heaton 1997).

Approximately 0.25 g of feces were used for DNA extraction with the DNeasy PowerLyzer PowerSoil Kit (Qiagen, ref. QIA12855) following manufacturer's instructions, and including a bead beating step to facilitate cell lysis. Quality control of genomic DNA was performed with a NanoDrop ND‐100 spectrophotometer (NanoDrop Technologies, DE, USA) to check the concentration and quality of the samples.

### 2.3 16S rRNA (V3‐V4) Library Preparation and Sequencing

Two samples of bacterial mock communities (HM‐276D and HM‐277D, from BEI Resources of the Human Microbiome Project (Peterson et al. 2009)) were included as positive controls for sequencing, each of them with ribosomal operons from genomic DNA of 20 bacterial species. These samples were processed as all other samples. HM‐276D has an even number of ribosomal DNA per species (1,000,000 copies per organism per µL), while HM‐277D has a variable number of operons (from 10,000 to 10,000,000 per organism per µL).

Amplification of 16S rRNA V3‐V4 region was performed as previously described (Willis et al. 2018), with V3‐V4‐F and V3‐V4‐R primers containing a 1–4 bp heterogeneity spacer to minimize the effects of low sequence diversity. Supporting Table 2 shows PCR conditions and primers.

A 10% of PhIX control libraries were included in our final libraries to raise the variety of sequencing reads. A pair‐end reads sequencing of 300 bp length in an Illumina MiSeq (Illumina San Diego, CA, USA,) using v3 chemistry, and a loading concentration of 15 pM was performed.

Throughout the process, negative controls of sample collection buffer, DNA extraction, and PCR were included. All were undetectable on gel visualization or Bioanalyzer analysis, in contrast to DNA from fecal samples which were always visible. We sequenced two of these negative controls.

Sequences have been submitted to the NCBI Sequence Read Archive (SRA) and can be accessed under the BioProject ID PRJNA918337.

### 2.4 Sequences Quality Trimming, Taxonomic Assignment, and Diversity Analyses

Data was preprocessed using the DADA2 pipeline (v. 1.24.0) (Callahan et al. 2016) obtaining 4837 amplicon sequence variants (ASVs) (Supporting File asvs.rds). The function filterAndTrim was applied to remove the low‐quality reads, in particular truncLen was set at 260 and 200 for forward and reverse reads, respectively and maxEE at 2 for both reads. The other parameters were left with default values. Subsequent steps in the pipeline were conducted following the guidelines set by the pipeline authors. The database used for the taxonomy assignment was SILVA v138 (Yilmaz et al. 2014).

The phylogenetic tree was obtained with the function AlignSeqs from the R package DECIPHER (v. 3.0.0) and the package phangorn (v. 2.12.1) with the functions NJ, pml and optim.pml. To root the tree we used the function midpoint.root from the phytools (v. 2.3.0) R package (Revell 2024). The resulting tree and ASV counts, along with sociodemographic data from each child, were stored and analyzed using the phyloseq package (v. 1.40.0) (McMurdie and Holmes 2013). Before proceeding with the taxa filtering step we checked if the taxa observed in the mock communities, are consistent with what is expected. In general, the observed composition was consistent with the expected one, with a couple of exceptions (Supporting Information S1: Figure S1). In the mock HM‐276, the phylum Campylobacterota is present in the observed composition but it was not expected. This phylum was not present in our data so it is expected to not affect the analysis. Regarding the mock HM‐277 from plate 2, the Actinobacteriota phylum was more abundant than expected. Therefore, from further analysis, we discarded the results if anything significant was identified in the mentioned phylum. Proceeding with the taxa filtering step, we discarded those taxa present only in the negative controls, taxa with < 100 reads and those found in less than two samples. In addition, samples with < 1000 total reads were removed. These correspond to two ESBL‐Es carriage positive samples.

Taxa relative abundance (values ranging between 0 and 1) was calculated at each taxonomic level by normalizing ASV counts per sample. A centered‐log ratio (clr) transformation of the data was applied to normalize raw ASV counts, normalizing them by the geometric mean of the taxa within each sample. This transformation was done by using the microbiome R package (v. 1.18.0) (Lahti and Shetty 2022). To be sure that there was not a significant difference in the number of reads among ESBL‐E carriers and non‐carriers, we performed a Wilcoxon rank‐sum test (Supporting Information S1: Figure S2, Supporting File sample_reads_count.csv) which did not show any influence of the variable on the data.

Different α‐ (within samples) and β‐diversity (between samples) measures were calculated using the Phyloseq and microbiome (v. 1.18.0) (Lahti and Shetty 2022) and phytools R packages (Revell 2024). Alpha diversity included four indices: the observed number of species, Shannon, Simpson, and Faith's PD. To ensure that the alpha‐diversity indices were not influenced by the filtering, we performed the calculation also with the unfiltered phyloseq object and with rarefied data (Supporting Information S1: Figures S3 and S4). Data rarefaction was performed after checking the read counts per sample and removing the two samples with fewer than 1000 reads. The rarefaction was implemented using the rarefy_even_depth function from the phyloseq package, setting the sample. size parameter to 8000 to retain all samples (minimum read count per sample = 8469). We set the rngseed to 123 and performed the rarefaction without replacement, leaving the rest of the function parameters at their default values. For beta‐diversity metrics, Aitchison distances were computed by calculating Euclidean distances on clr‐transformed raw ASV counts. Representation of the Aitchison distance was performed in a multi‐dimensional scaling (MDS) method to visualize the sample clustering.

### 2.5 Quantitative PCR (qPCR)

Because in all but one ESBL‐Es carrier the resistant strain was identified as *Escherichia coli* (Supporting Information S1: Table S1), the abundance of this species was determined by quantitative PCR (qPCR) as described previously (Lopez‐Siles et al. 2014). To calculate relative abundance and to normalize data, total bacteria quantification was also performed as reported elsewhere (Lopez‐Siles et al. 2014). Primers, probes, standards for quantification (16S rRNA gene inserted in a pCR4‐TOPO cloning plasmid and linearized with *Not*I), and PCR conditions are detailed in Supporting Information S1: Table S2. Samples were run in a 7500 Real Time PCR (Applied Biosystems) system in duplicate. In each run standard curves, non‐template controls and inhibition controls were included. Relative abundance data are given as the log10 of the ratio between *E. coli* 16S rRNA gene copies and millions of total bacterial 16S rRNA genes detected in the same sample.

### 2.6 Statistics

Normality and homoscedasticity were assessed using Shapiro‐Wilk and Levene's test, respectively. Sociodemographic differences by ESBL‐Es carriage were analyzed using a *t*‐test or Chi‐square test, as appropriate.

Alpha diversity was compared using the Wilcoxon rank‐sum test, also known as Mann‐Whitney U test. Beta diversity (weighted Unifrac distances and Aitchison distances) was evaluated via Permutational Multivariate Analysis of Variance (PERMANOVA) considering gender and age as covariates, and the sequencing run as strata (random effect). The PERMANOVA test was applied using the adonis2 R function from the vegan package (v. 2.6‐8) (Oksanen et al. 2022). Weighted UniFrac distances were calculated using the UniFrac function from the phyloseq package.

Differential abundance of taxa b was performed on clr‐transformed data with two approaches: the Mann–Whitney U test using the compare_means function from the ggpubr package (v. 0.6.0) (Wickham 2025) and correcting for multiple testing correction with the false‐discovery rate (FDR) method; and the generalized linear models using the glmer function from the lme4 R package (v. 4.2.1) (Bates et al. 2015), setting gender, age and school as fixed effects and the run as the batch effect. Multiple testing correction was performed applying the FDR method with the p.adj function from the stat R package (v. 4.4.1).

Relative abundance of *E. coli* in ESBL‐Es carriers and non‐carriers was compared using Mann‐Whitney *U* test, and differences by type of ESBL enzyme were assessed using Kruskal‐Wallis test followed by Dunn's Multiple Comparison test.

Statistical analyses were carried out in both IBM SPSS statistics version 15.0 and GraphPad Prism 5 v 5.01 software and by using the R programming language version 4.2.1 (Team 2022). Statistical significance was set at *p* ≤ 0.05.

## 3 Results

### 3.1 Characteristics of ESBL‐E Carriers and Non‐Carriers

Participant characteristics are summarized in Table 1 and data from the entire cohort have been previously reported (López‐Siles et al. 2023). ESBL‐Es carriers and non‐carriers were confirmed as age and gender matched (*p* ≥ 0.871). No statistically significant differences in all but two of the analyzed sociodemographic variables were found. A higher proportion of ESBL‐Es carriers had a relative with diarrhea the week before sampling (17%, *p* = 0.05) compared to non‐carriers. A higher number of pet (dog and/or cat) owners (26%, *p* = 0.049) was found in the carriers compared to the non‐carriers group.

**Table 1:** Characteristics of the total cohort of this study and by extended spectrum ß‐lactamase‐producing *Escherichia* (ESBL‐Es) carriage condition.

	*N* a		All subjects	ESBL‐Es non‐carriers	ESBL‐Es carriers	*p* value^b^	Test^b^
*n*			**48**	**24**	**24**		
Age							
	48	mean years ± SD	6.6 ± 2.2	6.5 ± 2.3	6.6 ± 2.3	0.871	^¥^
Gender	48	*n* males (%)	28 (58%)	14 (58%)	14 (58%)	1.000	^#^
School	48	*n* (%)				0.981	^#^
AG			10 (21%)	4 (17%)	6 (25%)		
AS			12 (25%)	7 (29%)	5 (21%)		
CC			4 (8.3%)	2 (8.3%)	2 (8.3%)		
LF			6 (12%)	3 (12%)	3 (812%)		
LV			8 (17%)	4 (17%)	4 (17%)		
MVM			8 (17%)	4 (17%)	4 (17%)		
Bristol scale	39	*n* (%)				0.929	^#^
2			7 (18%)	4 (23%)	3 (14%)		
3			4 (10%)	2 (12%)	2 (9.1%)		
4			12 (31%)	5 (29%)	7 (32%)		
5			11 (28%)	4 (23%)	7 (32%)		
6			5 (13%)	2 (12%)	3 (14%)		
WHO region of origin	46	*n* subjects (%)				0.489	^#^
America			2 (4.0%)	0 (0.0%)	2 (8.7%)		
Europe			44 (96%)	23 (100%)	21 (91%)		
Number of siblings	45	*n* subjects (%)					
None			10 (22%)	5 (21%)	5 (24%)	0.346	^#^
1			32 (71%)	18 (75%)	14 (67%)		
2			2 (4.4%)	0 (0.0%)	2 (9.5%)		
≥ 3			1 (2.2%)	1 (4.2%)	0 (0.0%)		
Diarrhea the past 7 days	47	*n* subjects (%)	1 (2.1%)	0 (0.0%)	1 (4.3%)	0.489	^#^
Family member with diarrhea the past 7 days	47		4 (8.5%)	0 (0.0%)	4 (17%)	**0.050**	^#^
Pets' owner	48	*n* subjects (%)				**0.049**	^#^
No pets			41 (85%)	23 (96%)	17 (74%)		
Cats			3 (6.3%)	0 (0.0%)	3 (13%)		
Dogs			3 (6.3%)	0 (0.0%)	3 (13%)		
Cats& Dogs			1 (2.1%)	1 (4.2%)	0 (0.0%)		
Source of drinking water	47	*n* subjects (%)				0.720	^#^
Tap water			33 (70%)	18 (75%)	15 (65%)		
Bottled water			2 (4.3%)	1 (4.2%)	1 (4.3%)		
Tap Water& Bottled water			8 (17%)	3 (12%)	5 (22%)		
Tap Water& Bottled water& spring/fountains			1 (2.1%)	1 (4.2%)	0 (0.0%)		
Tap Water& spring/fountains			3 (6.4%)	1 (4.2%)	2 (8.7%)		
Vegetables washing	47	*n* subjects (%)				0.212	^**#**^
Always			33 (70%)	19 (79%)	14 (61%)		
Habitually			14 (30%)	5 (21%)	9 (39%)		
Travel abroad in the past 6 months	46	*n* subjects (%)	6 (13%)	3 (12%)	3 (14%)	1.000	^**#**^
Travel to EU in the past 6 months	6	*n* subjects (%)	3 (50%)	1 (33%)	2 (67%)	1.000	^#^
Enteric protozoa	48	*n* carriers (%)	16 (33%)	6 (25%)	10 (42%)	0.359	^#^

^a^ Number of subjects out of the 48 participant that provided information for each variable is indicated. Percentages within each category have been calculated related to the number of individuals from which data was collected.

^b^ *p*‐value refers to comparison between ESBL‐E carriers and non‐carriers; ^¥^ *t*‐test, ^#^ Chi‐square test. Data had a normal distribution (Shapiro‐Wilk test, *p* ≥ 0.377 and variances were equal between groups (Levene's statistics, *p* = 0.951)

### 3.2 Sequencing Characteristics

High‐throughput sequencing analysis of bacterial 16S rRNA V3–V4 regions yielded a total of 758,939 high‐quality reads. An average of 16,498.67 ± 5,334.67 reads/sample were obtained from the 46 fecal samples included in the final analysis. Two ESBL‐Es carriers were removed by filtering out those samples with less than 1000 total reads (Supporting File metadata.csv). After quality filtering, 110 species were considered in the whole data set.

### 3.3 Alpha‐ and Beta‐Diversity of Gut Microbiota of ESBL‐E Carriers and Non‐Carriers

Alpha‐diversity was assessed using four indices (Figure 1A) and values of the indicators did not differ significantly between groups by ESBL‐Es carriage condition with filtered data (*p* ≥ 0.24). Also, with non‐filtered data (Supporting Information S1: Figure S3) and with rarefied data (Supporting Information S1: Figure S4), we did not identify any significant difference for the four indices (*p* ≥ 0.29 and *p* ≥ 0.097, respectively). No differences were observed in these indices between carriers with ESBL‐Es enzyme type SHV or CTX‐M (Supporting Information S1: Figure S5).

**Figure 1:**
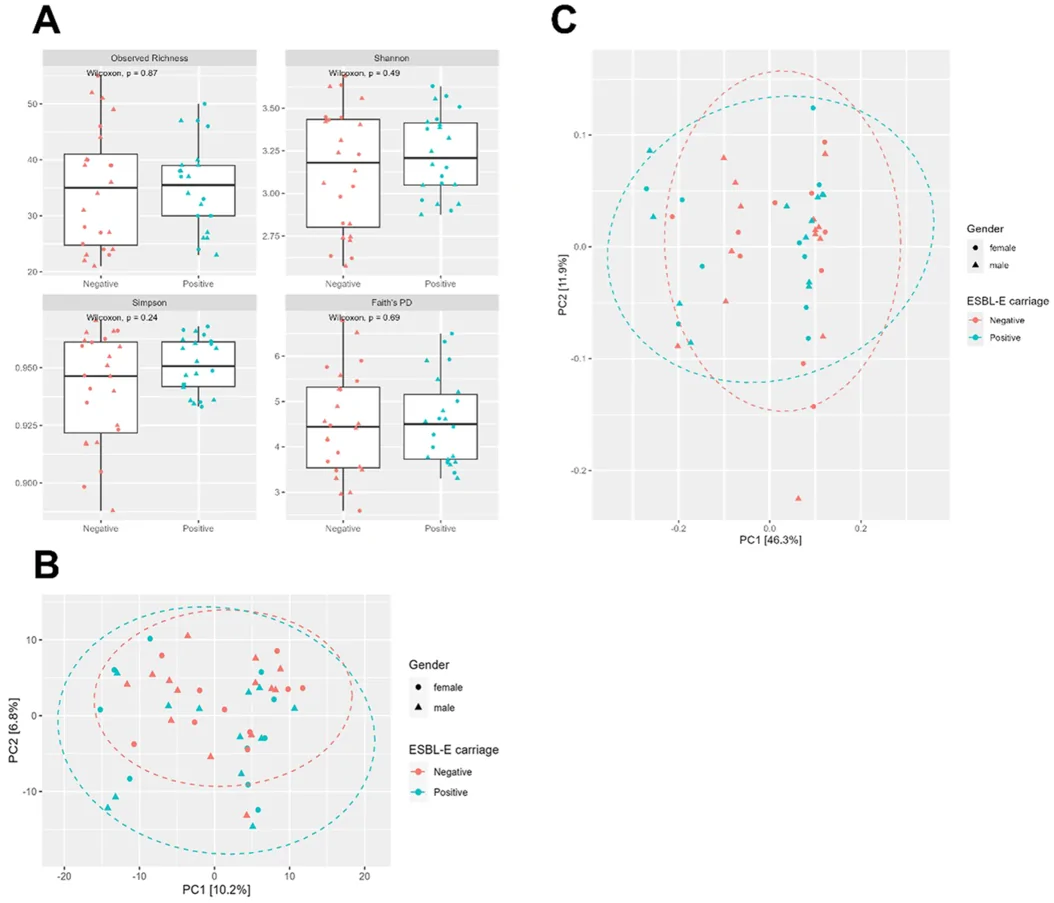
Alpha‐and Beta‐diversity analysis by ESBL‐Es carriage condition. (A) Boxplots representing α‐diversity indices: observed species (richness), Shannon, (diversity), Simpson (evenness), and Faith's PD. The plots represent the interquartile range (IQR) between the 25th, and 75th percentiles calculated for non–carriers of ESBL‐Es (negative) and carriers (positive) in fecal samples. The horizontal line in each box defines the median. Wilcoxon test used to check for significant differences among within‐sample taxonomic composition in children negative or positive for ESBL‐Es carriage. (B) Multidimensional scaling of Aitchison distances of microbial communities in the 46 samples (points). (C) Multidimensional scaling of Unifrac distances of microbial communities in the 46 samples (points).

The beta‐diversity analysis using Aitchison distances was not able to separate carriers and non‐carriers. The 2‐dimensional MDS visualization of the data showed no segregation or clustering based on ESBL‐Es carriage (Figure 1B). The first principal component (PC1) represented 10.2% while the second component (PC2) represented 6.8% of intersample variance, respectively. After performing PERMANOVA analysis to assess the effect of ESBL‐Es carriage on the overall composition, assigning gender and age as covariates and the sequencing run as random effect, being ESBL‐Es carrier or not was not identified as a source of variation among the samples. Similarly, for the weighted Unifrac distances (Figure 1C) no separatin of the samples on the basis of ESBL‐Es carriage was observed. The visual results were confirmed by the PERMANOVA analysis, where in both cases the variable was not identified as a source of variation among the samples. In particular, for the Aitchison distances, we obtained a p‐value of 0.3 and an R2 of 0.02, and for the weighted Unifrac the values correspond to 0.2 and 0.03, respectively. Within ESBL‐E carriers, no segregation was observed either regarding the ESBL enzyme type (Supporting Information S1: Figure S5). Altogether, these results suggest that there are no major differences in the overall composition of the gut microbiota in ESBL‐Es carriers compared to non‐carriers.

### 3.4 Differentially Abundant Bacteria in ESBL‐E Carriers and Non‐Carriers

We next investigated bacterial abundances (using centered log‐ratio–clr–transformation) to determine whether any specific taxa were differentially abundant between ESBL‐Es carriers and non‐carriers. The most abundant phyla in both ESBL‐Es carriers and non‐carriers were Firmicutes (64%), Actinobacteriota (21%), Bacteroidota (7.5%), and Verrucomicrobiota (5.7%). No significant differences (*p* ≥ 0.36) in clr‐abundance were found at this taxonomic level (Supporting Information S1: Figure S6).

The most abundant families regardless of the group were Lachnospiraceae (30%), Ruminococcaceae (13%) and Bifidobacteriaceae (11%). No significant differences were found at the family level either (*p* = 0.33, *p* = 0.43, and *p* = 0.41, respectively).

At the genus level, significant differences in the relative abundance were observed (Supporting Information S1: Figure S7). The genera *Blautia* was significantly lower in ESBL‐Es carriers (*p* = 0.01) and the genus *Coprococcus* was significantly higher (*p* = 0.04). At the species level (Figure 2A), *Blautia obeum* was significantly lower in ESBL‐Es carriers (*p* = 0.01) and *Coprococcus eutactus* was significantly higher (*p* = 0.02). To check that the findings were not driven by prevalence but by abundance, we also checked the genera relative abundances (Supporting Information S1: Figure S8A).

**Figure 2:**
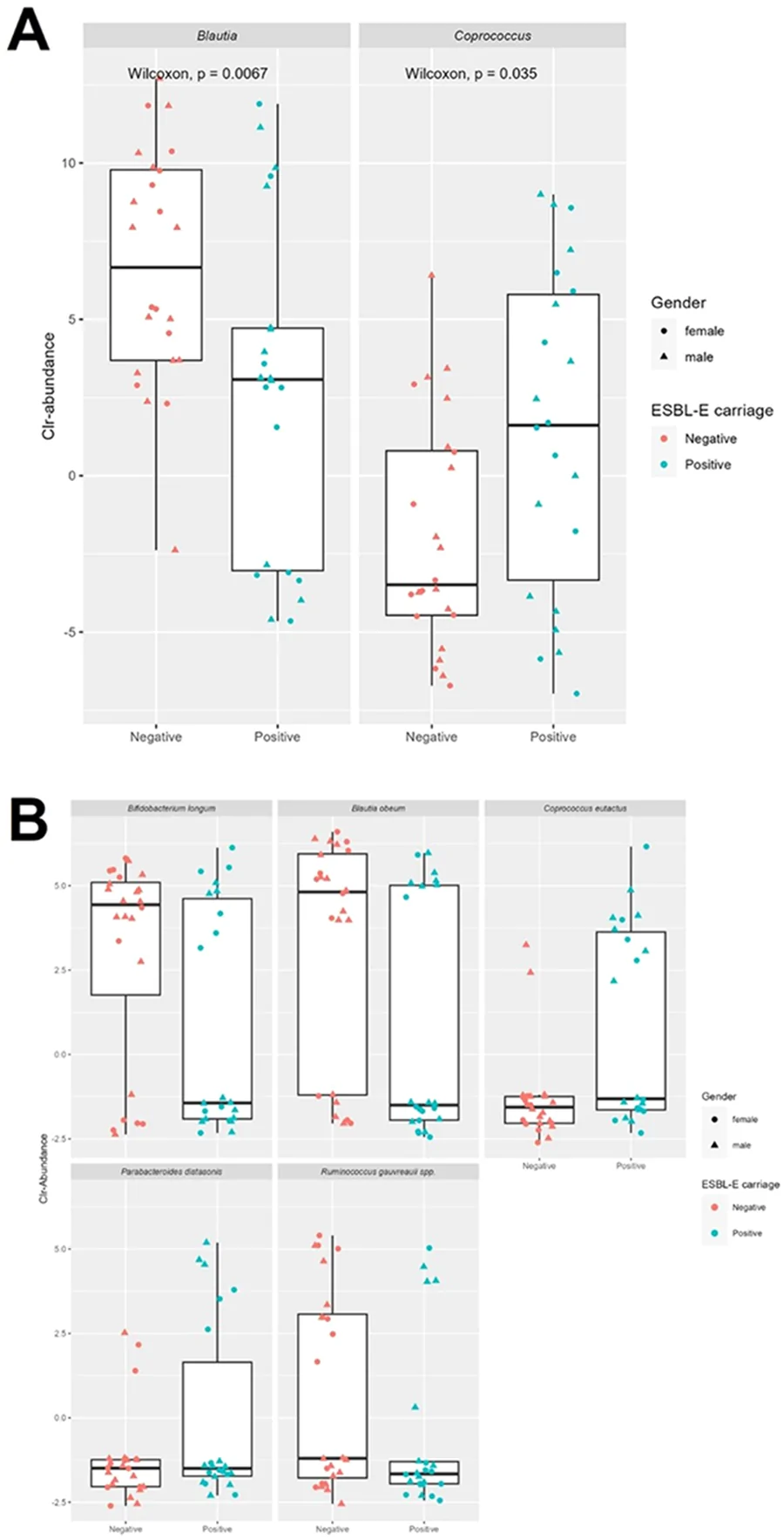
Differential taxa by ESBL‐Es carriage condition. Boxplots representing the centered log‐ratio (clr) transformation abundance of (A) the two genus and the (B) five species significantly different among samples positive and negative for carriage of ESBL‐Es after generalized linear models analysis. Wilcoxon test was used to compare between groups.

### 3.5 Linear Models to Identify Differentially Abundant Species

We used a complementary approach (generalized linear models) that allows controlling for gender, age and sequencing run effects, to investigate differential clr‐abundance levels at all taxonomic ranks. At family level, Tannerellaceae was more abundant in ESBL‐Es carriers compared to non‐carriers (*p* = 0.017, data not shown). Significant differences in abundance between groups were found for four genera: *Blautia* (*p* = 0.024) and *Ruminococcus* (*p* = 0.027) were more abundant in ESBL‐Es non‐carriers whereas, *Coprococcus* (*p* = 0.02) and *Parabacteroides* (*p* = 0.017) were more abundant in ESBL‐Es carriers. Accordingly, this approach identified five species whose relative abundance was significantly different between ESBL‐Es carriers and non‐carriers, namely: *B. obeum* (*p* = 0.047), *Bifidobacterium longum* (*p* = 0.019), *Ruminococcus gauvreauii* (*p* = 0.027), *C. eutactus* (*p* = 0.002) and *Parabacteroides distasonis* (*p* = 0.007) (Figure 2B). The first three species were identified as significantly more abundant in ESBL‐Es non‐carriers, and the last two in ESBL‐Es carriers. Also in this case, to check that the findings were due to actual abundance rather than prevalence, we plotted the five species' relative abundances (Supporting Information S1: Figure S8B) and appreciated that when in one group there were many points clustering near the zero, the same would happen in the other group, suggesting a difference not driven by detection.

Lastly, for selected bacterial taxa associated with changes in gut microbiota due to ESBL‐E carriage identified in previous studies (Gosalbes et al. 2016; Piewngam et al. 2019; Peng et al. 2021; Ducarmon et al. 2022), we reported a more detailed table (Table 2) regarding their abundances in total, in the two groups of interest and the associated *p*‐values and *p*‐adjusted values. No further differences were confirmed apart from those of the *Blautia* spp., *Cropococcus* spp., and *C. eutactus* (*p* ≤ 0.03). When applying FDR correction, none of the identified taxa mentioned above maintain a significant *p*‐value.

**Table 2:** Abundances of bacterial groups by extended spectrum ß‐lactamase‐producing *Escherichia* (ESBL‐Es) carriage condition.

Bacterial group^a^		All subjects	ESBL‐Es non‐carriers	ESBL‐Es carriers	*p* valuee	*p*‐adjusted values
*n*	*n* subjects	**46**	**24**	**22**		
Bacterial indicators^b^	(median ± SD)					
Ratios						
Firmicutes/Bacteroidetes		−0.57 ± 1.2	−0.46 ± 1.07	−0.63 ± 1.31	0.45	0.89
Firmicutes/Proteobacteria		−1.92 ± 520.82	−1.53 ± 6.58	−2.61 ± 752.39	0.53	0.89
* Akkermansia/Faecalibacterium*		‐0.18 ± 1.83	−0.36 ± 2.29	0.02 ± 1.17	0.92	0.92
* F. prausnitzii*/*Escherichia‐Shigella*		1 ± 2.32	1 ± 2.36	0.12 ± 2.31	0.66	0.89
Families						
Lachnospiraceae		3.21 ± 16.36	5.02 ± 16.03	0.97 ± 16.44	0.33	0.33
Genus						
* Anaerostipes*		1.96 ± 4.06	1.93 ± 4.28	1.49 ± 3.89	0.62	0.70
* Bifidobacterium*		9.41 ± 5.87	10.7 ± 4.91	6.89 ± 6.82	0.41	0.62
* Blautia*		4.62 ± 4.96	6.66 ± 3.79	3.07 ± 5.33	0.01	0.06
*Butyricicoccus*		−1.52 ± 2.1	−1.49 ± 2.22	−1.57 ± 2	0.89	0.89
* Coprococcus*		−0.91 ± 4.73	−3.49 ± 3.68	1.61 ± 5.18	0.03	0.16
* Escherichia/Shigella*		−1.49 ± 1.81	−1.49 ± 1.33	−1.5 ± 2.19	0.56	0.70
* Faecalibacterium*		2.29 ± 4.22	1.7 ± 3.61	3.3 ± 4.71	0.13	0.40
* Ruminococcus torques*		0.67 ± 4.38	−0.23 ± 4.32	1.12 ± 4.45	0.29	0.52
* Parabacteroides*		−2.77 ± 2.98	−2.78 ± 1.62	−2.75 ± 3.79	0.21	0.47
Species						
* Akkermansia muciniphila*		−1.27 ± 3.59	−1.2 ± 3.71	−1.37 ± 3.54	0.59	0.59
* Bacteroides uniformis*		−1.31 ± 2.55	−1.23 ± 2.56	−1.44 ± 2.59	0.23	0.45
* Collinsella aerofaciens*		4.71 ± 3.06	4.56 ± 3.25	4.86 ± 2.82	0.27	0.45
* Coprococcus eutactus*		−1.44 ± 2.47	−1.56 ± 1.36	−1.31 ± 2.91	0.02	0.11
* Eubacterium hallii*		4.59 ± 2.1	4.59 ± 2.38	4.54 ± 1.78	0.59	0.59

^a^ Abundances from clr‐transformed data (median ± SD) of bacterial groups associated to changes in gut microbiota due to ESBL‐Es carriage by previous studies (Gosalbes et al. 2016; Piewngam et al. 2019; Peng et al. 2021; Ducarmon et al. 2022). The Wilcoxon rank sum test *p*‐value and *p‐*adjusted value resulting from the comparison of ESBL‐Es carriers and non‐carriers are included in the last two columns.

^b^ The genus *Actinomyces*, *Bilophila*, *Corynebacterium*, *Desulfovibrio*, *Enterococcus*, *Lachnospira*, *Lactococcus*, *Marvinbryantia*, *Negativibacillus*, *Oscillospira*, *Phocea*, *Staphylococcus*, *Weissella*, and the sepcies *Pseudomonas fragi* were not present in the data.

### 3.6 *E. coli* Relative Abundance in Esbl‐Es Carriers and Non‐Carriers

*E. coli* abundance was not significantly different between ESBL‐Es carriers and non‐carriers (Figure 3A) even though children colonized by ESBL‐Es tend to feature higher abundance of *E. coli* than those not colonized (*p* = 0.119). No significant differences were observed between ESBL enzyme types (Figure 3B , *p*= 0.169) despite higher counts observed in children carrying an ESBL‐Es with the SHV enzyme type.

**Figure 3:**
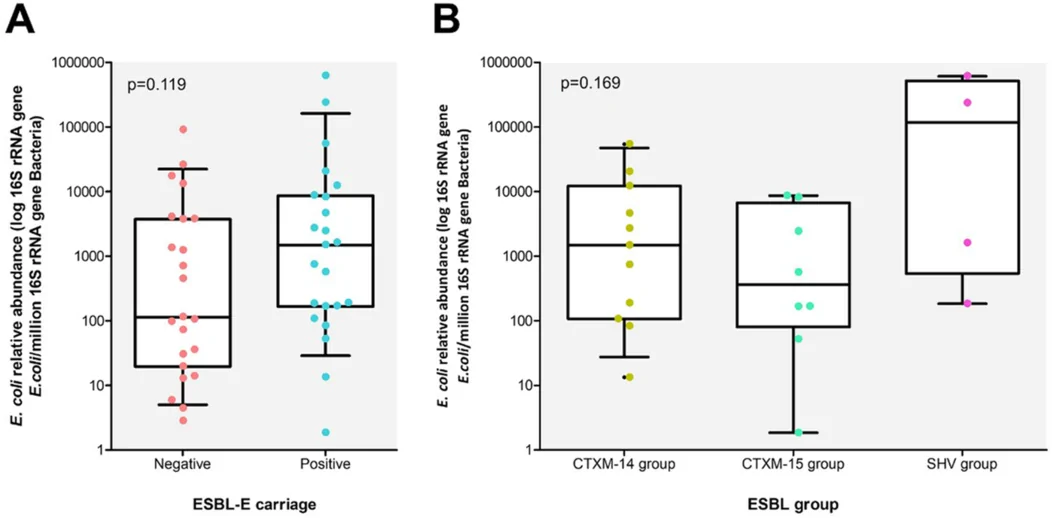
*Escherichia coli* relative abundances. Results categorized by ESBL‐E carriage condition (A) and by ESBL variant group within children colonized by ESBL‐E (B).

## 4 Discussion

In this study, the gut bacterial community of ESBL‐Es carriers and non‐carriers in healthy Spanish children was analyzed. To the best of our knowledge, this is the only study carried on community children to date, and among the few focused on an European cohort, assessing the impact of colonization by multidrug resistant microorganisms in the gut microbiota.

We observed no major differences in the overall structure and diversity of the bacterial gut microbiota. All calculated α‐diversity indices were similar between ESBL‐Es carriers and non‐carriers. This result agrees with three previous reports analyzing gut microbiota from adult ESBL‐E carriers and non‐carriers from French Guiana, Thailand, and Deutschland (Gosalbes et al. 2016; Piewngam et al. 2019; Ducarmon et al. 2022). In contrast, in international travelers from China a higher richness in Actinobacteria associated with a lower risk of ESBL‐E colonization (Peng et al. 2021) has been evidenced. Although this phylum is less represented in children than in adults (Ottman et al. 2012), in our cohort similar abundances were observed in ESBL‐Es carriers and non‐carriers. Similarly, β‐diversity analysis did not reveal a clustering of subjects by ESBL‐Es carriage condition. Overall, our results suggest that there are no major differences in the general composition of the microbiota and that the impact of ESBL‐E carriage may be constrained to certain taxa. This study is limited by factors known to influence gut microbiota which we did not control for (Rojo et al. 2017), and the limited number of subjects engaged might contribute to weakening statistical power and prevent reaching significance or clustering.

Despite the absence of global differences, several significant differences at lower taxonomical levels were identified. Specifically, five differentially abundant species were found after using generalized linear models. *Blautia obeum, B. longum* and *R. gauvreauii* were more abundant in non‐carriers, while *C. eutactus* and *P. distasonis* whose relative abundance was significantly higher in ESBL‐Es carriers. The differences concerning *B. obeum* and *C. eutactus* were also supported by simpler comparisons of clr‐based abundances using a Wilcoxon test. In previous studies on non‐Western populations, different species of *Bifidobacterium* spp. were identified as more abundant in ESBL‐E non‐carriers (Peng et al. 2021), which matches our findings. Bifidobacteria have been associated with the production of health‐promoting metabolites or the protection against infection (Arboleya et al. 2016).

Regarding *C. eutactus*, controversial results have been previously reported, because an increase in this species has been evidenced in non ESBL‐E carriers (Gosalbes et al. 2016) and in carriers (Piewngam et al. 2019). Our finding concurs with the latter. Differences in baseline microbiota of participants between cohorts may exist and explain inconsistencies among different studies, as Gosalbes and colleagues enrolled an isolated Amerindian community (Gosalbes et al. 2016). *C. eutactus* is an anaerobic butyrate‐producer bacteria, known for exerting beneficial effects on the intestinal homeostasis, like supporting gut barrier function (Guo et al. 2020). Our study design does not allow us to determine if the increase in this species is a cause or a consequence of ESBL‐Es colonization, but according to present knowledge of this species role, this change may not exert a negative effect on host wellbeing.

Furthermore, we found differences in relative abundance of three species namely *B. obeum, R. gauvreauii* and *P. distasonis*, which none of the previous studies observed. Further corroboration studies are required to confirm if those could be specific alterations in children. In line with this hypothesis, these species have been associated with dysbiosis in children suffering different conditions (Fan et al. 2022; Gozalbo‐Rovira et al. 2021; Matos et al. 2021). A limitation of the present study is that we cannot decipher whether the differences observed are due to antibiotic treatment, hospitalization, BMI, intestinal disorders/history of gastrointestinal disease, diet, ethnicity or other factors known to influence gut microbiota composition (Rojo et al. 2017) because this information was not registered in the questionnaire.

## 5 Conclusions

This study reveals differences in gut microbiota species abundance among community children due to carriage of ESBL‐Es, a group of antibiotic‐resistant bacteria considered worrisome by WHO. Although after multiple testing correction, none of the identified species remain significantly different, likely due to limited sample size, the initial findings deserve further investigation. Future studies should aim to validate these preliminary observations with larger cohorts.

Additionally, studies should explore whether enhancement of species predominant in non‐carriers and/or isolation of molecules from these species may help in finding strategies to prevent carriage of ESBL‐Es. It would be also of interest to explore whether the observed alterations in the gut microbiota of ESBL‐Es carriers have an impact on the wellbeing of children.

## Author Contributions

**Martina Cardinali‐Benigni:** data curation, formal analysis, investigation, software, visualization, writing – original draft preparation. **Olfat Khannous‐Lleiffe:** formal analysis, investigation, software, visualization, writing – review & editing. **Ester Saus:** formal analysis, investigation, writing – review & editing. **David Carmena:** conceptualization, data curation, funding acquisition, resources, supervision, writing – review & editing. **Michael J. McConnell:** conceptualization, funding acquisition, resources, supervision, writing – review & Editing. **Toni Gabaldón:** conceptualization, data curation, funding acquisition, methodology, resources, software, supervision, visualization, writing – original draft preparation, writing – review & editing. **Mireia López‐Siles:** conceptualization, data curation, formal analysis, funding acquisition, investigation, methodology, project administration, resources, supervision, visualization, writing – original draft preparation, writing – review & editing. All authors read and approved the final manuscript.

## Ethics Statement

Before enrollment of volunteers, written informed consent to participate in the study was signed from parents/legal guardians of participating schoolchildren, in accordance with the Declaration of Helsinki. The Ethics Committee of the Health Institute Carlos III approved all study procedures, informed consent forms, and epidemiological questionnaires on 17 September 2018 (reference number CEI PI 17_2017‐v3).

## Consent

The authors have nothing to report.

## Conflicts of Interest

M.J.M. is founder and stockholder of the biotechnology spin‐off company Vaxdyn, which develops vaccines for infections caused by MDR bacteria. Vaxdyn had no role in the elaboration of this manuscript. The other authors declare no conflicts of interest.

## Supporting information


**Supplementary Table 1**: Information of ESBL‐E strains obtained from ESBL‐E carriers. Age and gender of children of isolation source, sequence type, type of extended spectrum ß‐lactamase (ESBL) enzymes and antimicrobial resistance phenotype. Black cells indicate resistant, grey intermediate and white sensitive for a given antibiotic. **Supplementary Table 2**: Primers used in this study and PCR conditions. **Supplementary Figure 1.Comparison of theknown** taxa included in the mock communities HM‐276 and HM‐277 and that observed after analysis. **Supplementary Figure 2.** Distribution of sequencing reads in ESBL‐Es carriers and non carriers. **Supplementary Figure 3:** Alpha‐diversity analysis by ESBL‐Es carriage condition using non‐filtered data. Boxplots represent α‐diversity indices: observed species (richness), Shannon, (diversity), Simpson (evenness), and Faith's PD. The plots represent the interquartile range (IQR) between the 25th, and 75th percentiles calculated for non–carriers of ESBL‐Es (negative) and carriers (positive) in fecal samples. The horizontal line in each box defines the median. Wilcoxon test used to check for significant differences among within‐sample taxonomic composition in children negative or positive for ESBL‐Es carriage. **Supplementary Figure 4:** Alpha‐diversity analysis by ESBL‐Es carriage condition using rarefied data. Boxplots represent α‐diversity indices: observed species (richness), Shannon, (diversity), Simpson (evenness), and Faith's PD. The plots represent the interquartile range (IQR) between the 25th, and 75th percentiles calculated for non–carriers of ESBL‐Es (negative) and carriers (positive) in fecal samples. The horizontal line in each box defines the median. Wilcoxon test used to check for significant differences among within‐sample taxonomic composition in children negative or positive for ESBL‐Es carriage. **Supplementary Figure 5:** Alpha‐ and beta‐diversity analyses of the bacterial community in children ESBL‐E carriers by type of enzyme. **Supplementary Figure 6:** Boxplots showing the centered log‐ratio (clr) abundance at the phylum level of the bacterial community present in the analyzed stool samples. Wilcoxon test was used to compare and eventually find significant differences in the abundances among samples positive and negative for ESBL‐producing bacteria. The points' shape represents the samples' gender (circle for female and triangle for male). The color represents the different phyla. **Supplementary Figure 7:** Relative abundances at the genus levels of the bacterial communities present in fecal samples of children negative and positive for ESBL‐E carriage. On the *x* axis each column corresponds to a sample, on the *y* axis is reported the value of the relative abundances. **Supplementary Figure 8:** Differential taxa by ESBL‐Es carriage condition. asvs. metadata. sample_reads_counts.

Supporting Information

Supporting Information

Supporting Information

## Data Availability

Data available on request due to provacy/ethical restrictions.
