# Supplementary material for: Identification of a Differential Microbiological Signature of Extended‐Spectrum ß‐Lactamases Producing Escherichia Carriers in Spanish Community Children
Source: Microbiologyopen. 2025 Oct 29;14(6):e70077. doi: 10.1002/mbo3.70077 (PMC13750485; doi:10.1002/mbo3.70077)
Supplement: Supplementary file 1 [file MBO3-14-e70077-s001.docx]

**SUPPLEMENTAL MATERIALS**

**Supplementary Table 1**. Information of ESBL-E strains obtained from ESBL-E carriers. Age and gender of children of isolation source, sequence type, type of extended spectrum ß-lactamase (ESBL) enzymes and antimicrobial resistance phenotype. Black cells indicate resistant, grey intermediate and white sensitive for a given antibiotic.

|  |  |  |  |  |  |  |  |  | **Antimicrobials resistance^a^** | | | | |  |  |  |  |  |  |  |  |  |  |  |  |  |  |  |  |  |  |  |
| --- | --- | --- | --- | --- | --- | --- | --- | --- | --- | --- | --- | --- | --- | --- | --- | --- | --- | --- | --- | --- | --- | --- | --- | --- | --- | --- | --- | --- | --- | --- | --- | --- |
| **Isolate** | **Age**  **(years)** | **Gender** | **Species** | **ST** |  | **ESBL** | |  | **Beta-lactams** | | | | | | | | | | | | |  | **Quinolones** | | |  | **Others** | | | | | |
|  |  |  |  |  |  | **Enzyme group** | **Enzyme type** |  | **AMP** | **TIC** | **P** | **MEC** | **AMC** | **FOX** | **CRM** | **CFE** | **FOT** | **CPD** | **CAZ** | **FEP** | **AZT** |  | **NXN** | **CIP** | **LEV** |  | **AK** | **GEN** | **TOB** | **NIT** | **TRI** | **TRS** |
| 1 | 6 | F | *E. coli* | 48 |  | CTX-M-9 | CTX-M-9 |  | x | x | x | x |  |  | x | x | x | x | x | x | x |  | x | x | x |  |  | x | x |  | x | x |
| 2 | 9 | M | *E. coli* | 58 |  | CTX-M-9 | CTX-M-14 |  | x | x | x |  |  |  | x | x | x | x |  | x | x |  |  |  |  |  |  |  |  |  | x | x |
| 3 | 5 | M | *E. coli* | 40 |  | CTX-M-9 | CTX-M-14 |  | x | x | x |  | x |  | x | x | x | x | x | x | x |  |  |  |  |  |  |  |  |  |  |  |
| 4 | 8 | M | *E. coli* | 69 |  | CTX-M-1 | CTX-M-15 |  | x | x | x |  |  |  | x | x | x | x | x | x | x |  |  |  |  |  |  |  |  |  | x | x |
| 5 | 8 | M | *E. marmotae* | None |  | SHV | SHV-12 |  | x | x | x |  |  |  | x | x | x | x | x | x | x |  |  |  |  |  |  |  |  |  |  |  |
| 6 | 8 | M | *E. coli* | 10 |  | SHV | SHV-12 |  | x | x | x |  |  |  |  | x | x | x | x |  | x |  |  |  |  |  |  |  |  |  | x | x |
| 7 | 5 | F | *E. coli* | 131 |  | CTX-M-9 | CTX-M-27 |  | x | x | x |  |  |  | x | x | x | x | x | x | x |  | x |  |  |  |  | x | x | x | x | x |
| 8 | 10 | F | *E. coli* | 58 |  | CTX-M-9 | CTX-M-14 |  | x | x | x |  |  |  | x | x | x | x |  | x | x |  |  |  |  |  |  |  |  |  |  |  |
| 9 | 6 | F | *E. coli* | 69 |  | CTX-M-1 | CTX-M-15 |  | x | x | x |  |  |  | x | x | x | x | x | x | x |  |  |  |  |  |  |  |  |  |  |  |
| 10 | 8 | M | *E. coli* | 10 |  | CTX-M-1 | CTX-M-32 |  | x | x | x |  |  |  | x | x | x | x | x | x | x |  |  |  |  |  |  |  |  |  |  |  |
| 11 | 3 | M | *E. coli* | 1684 |  | CTX-M-9 | CTX-M-14 |  | x | x | x |  |  |  | x | x | x | x |  | x | x |  |  |  |  |  | x |  |  |  |  |  |
| 12 | 8 | F | *E. coli* | 58 |  | CTX-M-9 | CTX-M-14 |  | x | x | x |  |  |  | x |  | x | x |  |  |  |  |  |  |  |  |  |  |  |  | x | x |
| 13* | 3 | M | *E. coli* | 10 |  | SHV | SHV-12 |  | x | x | x |  |  |  | x | x | x | x | x |  | x |  | x | x | x |  |  |  |  |  |  |  |
| 14* | 3 | M | *E. coli* | 164 |  | SHV | SHV-12 |  | x | x | x |  |  |  | x | x | x | x | x |  | x |  |  |  |  |  |  |  |  |  |  |  |
| 15 | 10 | M | *E. coli* | 90 |  | SHV | SHV-12 |  | x | x | x |  |  |  |  |  | x | x |  |  |  |  |  |  |  |  |  |  |  |  |  |  |
| 16 | 11 | M | *E. coli* | 131 |  | CTX-M-1 | CTX-M-15 |  | x | x | x |  | x |  | x | x | x | x |  |  | x |  | x | x | x |  |  |  | x |  | x | x |
| 17 | 4 | M | *E. coli* | 752 |  | CTX-M-9 | CTX-M-14 |  | x | x | x |  |  |  | x | x | x | x |  | x | x |  | x |  |  |  |  |  |  |  |  |  |
| 18 | 3 | M | *E. coli* | 131 |  | CTX-M-1 | CTX-M-15 |  | x | x | x |  | x |  | x | x | x | x |  |  |  |  | x | x | x |  | x |  | x |  | x | x |
| 19 | 5 | F | *E. coli* | 10 |  | CTX-M-1 | CTX-M-32 |  | x | x | x |  |  |  | x | x | x | x | x | x | x |  |  |  |  |  |  |  |  |  |  |  |
| 20 | 6 | M | *E. coli* | 617 |  | CTX-M-1 | CTX-M-15 |  | x | x | x |  |  |  | x | x | x | x | x | x | x |  | x | x | x |  |  |  | x |  | x | x |
| 21 | 7 | F | *E. coli* | 131 |  | CTX-M-9 | CTX-M-27 |  | x | x | x |  |  |  | x | x | x | x | x | x | x |  | x | x | x |  |  |  |  |  | x | x |
| 22 | 7 | F | *E. coli* | 10 |  | CTX-M-9 | CTX-M-14 |  | x | x | x |  |  |  | x | x | x | x | x | x |  |  |  |  |  |  |  |  |  |  |  |  |
| 23 | 6 | M | *E. coli* | 90 |  | CTX-M-9 | CTX-M-27 |  | x | x | x |  |  |  | x | x | x | x |  |  | x |  | x | x | x |  |  |  |  |  | x | x |
| 24 | 6 | F | *E. coli* | 10 |  | CTX-M-1 | CTX-M-32 |  | x | x | x |  |  |  | x | x | x | x | x | x | x |  |  |  |  |  |  |  |  |  |  |  |
| 25 | na | F | *E. coli* | 93 |  | SHV | SHV-12 |  | x | x | x |  |  |  |  | x | x | x | x |  | x |  |  |  |  |  |  |  |  |  | x | x |

*Isolates from the same subject

n.a.: not available

^a^ Compounds abbreviations: AMP, Ampicillin; TIC, Ticarcillin; P, Piperacillin; MEC, Mecillinam; AMC, Amoxicillin-Clavulanic; FOX, Cefoxitin; CRM, Cerfurixime; CFE, Cefixime; FOT, Cefotaxime; CPD, Cefpodoxime; CAZ, Ceftazidime; FEP, Cefepime; AZT, Aztreonam; AK, Amikacin; GEM, Gentamicin; TOB, Tobramycin; NXN, Norfloxacin; CIP, Ciprofloxacin; LEV, Levofloxacin; NIT, Nitrofurantoin; TRI, Trimethoprim; TRS, Trimethoprim-Sulfamethoxazol. Other compounds tested but with nonsusceptibility for all strains were: P/T, Piperacillin-Tazobactam; FOTC, Cefotaxime-Clavulanic; CAZC, Ceftazidime-Clavulanic; ERT, Ertapenem; IMI, Imipenem; MER, Meropenem; NAL, Nalidixic acid; FOS, Fosfomycin, COL, Colistin, TGC, Tigecycline. Abbreviations in italics indicate intermediate susceptibility.

**Supplementary Table 2**. Primers used in this study and PCR conditions.

| **Target** | **Primer and probes^a^** | **Sequence (5’-3’)** | **Use** | **Cycles^b^** | **Denaturing (ºC; sec)^c^** | **Annealing (ºC; sec) ^c^** | **Extension**  **(ºC; sec) ^c^** | **Ref** |
| --- | --- | --- | --- | --- | --- | --- | --- | --- |
| *bla*_SHV_ | SHVM-F | AACGGAACTGAATGAGGCG | ESBL gene identification | 25 | 95; 45 | 65; 45 | 72; 45 | [1] |
|  | SHVM-R | TCCACCATCCACTGCAGCAGCT |  |  |  |  |  |  |
| *bla*_CTX-M-14_ | CTXM14M-F | TACCGCAGATAATACGCAGGTG | ESBL gene identification | 25 | 95; 45 | 65; 45 | 72; 45 |  |
|  | CTXM14M-R | CAGCGTAGGTTCAGTGCGATCC |  |  |  |  |  |  |
| *bla*_CTX-M-15_ | CTXM15M-F | AATCACTGCGCCAGTTCACGCT | ESBL gene identification | 25 | 95; 45 | 65; 45 | 72; 45 |  |
|  | CTXM15M-R | GAACGTTTCGTCTCCCAGCTGT |  |  |  |  |  |  |
| 16S rRNA  (V3-V4 region) | V3-V4-F+1 | TCGTCGGCAGCGTCAGATGTGTATAAGAGACAGNCCTACGGGNGGCWGCAG | Microbial community analysis | 25 | 95; 30 | 55; 30 | 72; 30 | [2] |
|  | V3-V4-F+2 | TCGTCGGCAGCGTCAGATGTGTATAAGAGACAGNNCCTACGGGNGGCWGCAG |  |  |  |  |  |  |
|  | V3-V4-F+3 | TCGTCGGCAGCGTCAGATGTGTATAAGAGACAGNNNCCTACGGGNGGCWGCAG |  |  |  |  |  |  |
|  | V3-V4-F+4 | TCGTCGGCAGCGTCAGATGTGTATAAGAGACAGNNNNCCTACGGGNGGCWGCAG |  |  |  |  |  |  |
|  | V3-V4-R+1 | GTCTCGTGGGCTCGGAGATGTGTATAAGAGACAGNGACTACHVGGGTATCTAATCC |  |  |  |  |  |  |
|  | V3-V4-R+2 | GTCTCGTGGGCTCGGAGATGTGTATAAGAGACAGNNGACTACHVGGGTATCTAATCC |  |  |  |  |  |  |
|  | V3-V4-R+3 | GTCTCGTGGGCTCGGAGATGTGTATAAGAGACAGNNNGACTACHVGGGTATCTAATCC |  |  |  |  |  |  |
|  | V3-V4-R+4 | GTCTCGTGGGCTCGGAGATGTGTATAAGAGACAGNNNNGACTACHVGGGTATCTAATCC |  |  |  |  |  |  |
| DNA IAC | IAC F | TACGGATGAGGAGGACAAAGGA | Quantitative PCR | 40 | 95; 15 | 60;60 | | [3] |
|  | IAC R | CACTTCGCTCTGATCCATTGG |  |  |  |  |  |  |
|  | **IAC PR** | **Cy5-CGCCGCTATGGGCATCGCA-BHQ-2** |  |  |  |  |  |  |
| Bacteria | F_Bact_1369 | CGGTGAATACGTTCCCGG | Quantitative PCR | 50 | 95; 15 | 60;60 | | [4] |
|  | R_Prok_1492 | TACGGCTACCTTGTTACGACTT |  |  |  |  |  |  |
|  | **P_TM_1389F** | **6FAM-CTTGTACACACCGCCCGTC-TAMRA** |  |  |  |  |  |  |
| *E. coli* | E.coli 395 F | CATGCCGCGTGTATGAAGAA | Quantitative PCR | 40 | 95; 15 | 60;60 | | [5] |
|  | E.coli 490 R | CGGGTAACGTCAATGAGCAAA |  |  |  |  |  |  |
|  | **E.coli 437 PR** | **6FAM-TATTAACTTTACTCCCTTCCTCCCCGCTGAA-TAMRA** |  |  |  |  |  |  |

^a^ Probe sequences are in bold. Cy5 (Cyanine 5), BHQ-2 (Black Hole Quencher 2), FAM^TM^ (6-carboxyfluorescin), TAMRA^TM^ (tetramethylrhodamin).

^b^ IAC, Internal Amplification Control; DNA IAC sequence: 5’-TACGGATGAGGAGGACAAAGGACGCCGCTATGGGCATCGCACCAATGGATCAGAGCGAAGTG-3’.

^c^ Program for conventional PCR for *bla* allele analysis included an initial denaturing step at 95ºC for 5 min and a final elongation step at 72ºC for 10 min. For all quantitative PCR, an initial step at 50ºC during 2 min was performed for amperase treatment. Also an initial denaturing step was set at 95ºC for 10 min. In quantitative PCR, annealing and extension steps were performed simultaneously.

For the 16S rRNA, PCR included an initial denaturing step at 95ºC for 3 min and a final elongation step at 72ºC for 5 min.

**Supplementary Figure 1.** Comparison of the known taxa included in the mock communities HM-276 and HM-277 and that observed after analysis

**Supplementary Figure 2.** Distribution of sequencing reads in ESBL-Es carriers and non carriers.

**Supplementary Figure 3.** Alpha-diversity analysis by ESBL-Es carriage condition using non-filtered data. Boxplots represent α-diversity indices: observed species (richness), Shannon, (diversity), Simpson (evenness), and Faith’s PD. The plots represent the interquartile range (IQR) between the 25th, and 75th percentiles calculated for non–carriers of ESBL-Es (negative) and carriers (positive) in fecal samples. The horizontal line in each box defines the median. Wilcoxon test used to check for significant differences among within-sample taxonomic composition in children negative or positive for ESBL-Es carriage.

**Supplementary Figure 4.** Alpha-diversity analysis by ESBL-Es carriage condition using rarefied data. Boxplots represent α-diversity indices: observed species (richness), Shannon, (diversity), Simpson (evenness), and Faith’s PD. The plots represent the interquartile range (IQR) between the 25th, and 75th percentiles calculated for non–carriers of ESBL-Es (negative) and carriers (positive) in fecal samples. The horizontal line in each box defines the median. Wilcoxon test used to check for significant differences among within-sample taxonomic composition in children negative or positive for ESBL-Es carriage.

**Supplementary Figure 5.** Alpha- and beta-diversity analyses of the bacterial community in children ESBL-E carriers by type of enzyme. **A.** Boxplots representing α-diversity indices: species observed (richness), Shannon, (diversity) and Simpson (evenness). The plots represent the interquartile range (IQR) between the 25th, and 75th percentiles calculated for ESBL-E carriers colonized by a strain with the CTX-M or SHV enzyme. The horizontal line in each box defines the median. Wilcoxon test used to check for significant differences among within-sample taxonomic composition in children according to enzyme type. **B.** Multidimensional scaling of Aitchison distances of microbial communities in the 22 samples of ESBL-E carriers. The shape corresponds to the gender and the ellipses and points’ color denote the sample from children with CTX-M (salmon) or SHV (light blue) ESBL enzyme.

**Supplementary Figure 6.** Boxplots showing the centered log-ratio (clr) abundance at the phylum level of the bacterial community present in the analyzed stool samples. Wilcoxon test was used to compare and eventually find significant differences in the abundances among samples positive and negative for ESBL-producing bacteria. The points’ shape represents the samples’ gender (circle for female and triangle for male). The color represents the different phyla.

**Supplementary Figure 7.**   Relative abundances at the genus levels of the bacterial communities present in fecal samples of children negative and positive for ESBL-E carriage. On the *x* axis each column corresponds to a sample, on the *y* axis is reported the value of the relative abundances.

**Supplementary Figure 8. Differential taxa by ESBL-Es carriage condition.** Boxplots representing the relative abundance of (**A**) the two genus and the (**B**) five species significantly different among samples positive and negative for carriage of ESBL-Es.

.

**Supplementary references**

[1] López-Siles, M., et al., Fecal carriage of extended-spectrum beta-lactamase-producing Enterobacterales in healthy Spanish schoolchildren. Front Microbiol, 2023. 14: p. 1035291. doi: 10.3389/fmicb.2023.1035291.

[2] Willis, J.R., et al., Citizen science charts two major "stomatotypes" in the oral microbiome of adolescents and reveals links with habits and drinking water composition. Microbiome, 2018. 6(1): p. 218. doi: 10.1186/s40168-018-0592-3.

[3] Lopez-Siles, M., et al., Mucosa-associated *Faecalibacterium prausnitzii* and *Escherichia coli* co-abundance can distinguish Irritable Bowel Syndrome and Inflammatory Bowel Disease phenotypes. International Journal of Medical Microbiology, 2014. 304(3-4): p. 464-475. doi: 10.1016/j.ijmm.2014.02.009.

[4] Furet, J.P., et al., Comparative assessment of human and farm animal faecal microbiota using real-time quantitative PCR. FEMS Microbiol Ecol, 2009. 68(3): p. 351-62. doi: 10.1111/j.1574-6941.2009.00671.x.

[5] Huijsdens, X.W., et al., Quantification of bacteria adherent to gastrointestinal mucosa by real-time PCR. J Clin Microbiol, 2002. 40(12): p. 4423-7. doi: 10.1128/JCM.40.12.4423-4427.2002.
